# Enhanced outcoupling in down-conversion white organic light-emitting diodes using imprinted microlens array films with breath figure patterns

**DOI:** 10.1080/14686996.2018.1551040

**Published:** 2018-11-29

**Authors:** Joo Won Han, Chul Woong Joo, Jonghee Lee, Dong Jin Lee, Jisoo Kang, Seunggun Yu, Woo Jin Sung, Nam Sung Cho, Yong Hyun Kim

**Affiliations:** a Department of Display Engineering, Pukyong National University, Busan, Republic of Korea; b Flexible Information Device Research Center, Electronics and Telecommunications Research Institute (ETRI), Daejeon, Republic of Korea; c Department of Creative Convergence Engineering, Hanbat National University, Daejon, Republic of Korea; d Robert Frederick Smith School of Chemical and Biomolecular Engineering, Cornell University, New York, NY, USA; e Department of Materials Science and Engineering, Yonsei University, Seoul, Republic of Korea

**Keywords:** Organic light-emitting diodes, down-conversion, light extraction, white OLED, outcoupling structures, 40 Optical, magnetic and electronic device materials, 201 Electronics / Semiconductor / TCOs, 204 Optics / Optical applications, organic light-emitting diodes

## Abstract

We demonstrate high-performance down-conversion microlens array (DC-MLA) films for white organic light-emitting diodes (OLEDs). The DC-MLA films are readily fabricated by an imprinting method based on breath figure patterns, which are directly formed on the polymer substrate with a novel concept. The DC-MLA films result in high-quality white light as well as enhanced light outcoupling efficiency for white OLEDs. The external quantum efficiency and power efficiency of OLEDs with DC-MLA films are increased by a factor of 1.35 and 1.86, respectively, compared to OLEDs without outcoupling films. Moreover, the white OLEDs with DC-MLA films achieve a high color-rendering index of 84.3. It is anticipated that the novel DC-MLA films fabricated by the simple imprinting process with breath figure patterns can contribute to the development of efficient white OLEDs.

## Graphical Abstract

**Figure:**
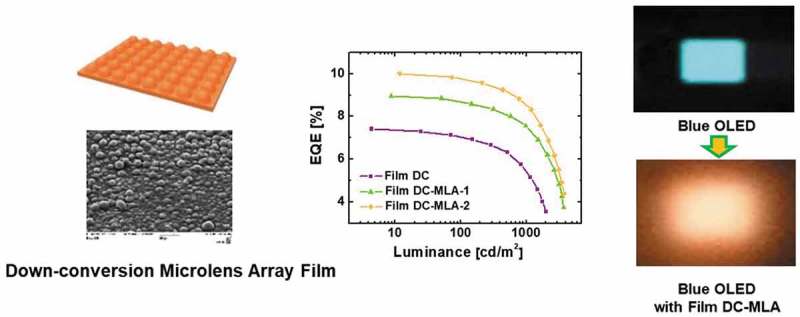

## 1. Introduction

White organic light-emitting diodes (OLEDs) have attracted considerable attention due to their important applications in flat panel displays and general lighting [1–3]. Their steadily improving brightness, efficiency, and stability have led to commercial products in recent years. The biggest challenge of conventional OLEDs is the limited light outcoupling efficiency about 20% while the internal quantum efficiency of OLEDs approaches 100%. The low light outcoupling efficiency is attributed to the trapped light in the substrate (substrate mode) and transparent electrodes/organic layers (waveguide mode) due to total internal reflection resulting from different refractive index of each layer [2,4].

In this respect, numerous light outcoupling techniques have been investigated to enhance the device efficiency. The index-matched hemisphere/microlens patterns or micro/nano-patterned device structures have been introduced to the extraction of surface plasmon polariton modes at the metallic cathode/organic interface [5,6]. To extract the waveguide mode, internal outcoupling structures such as low-index grids [7], light scattering layers [8,9], photonic crystals [10,11], corrugated structures [12–15], and high-index substrates [3,16] have been introduced. However, most internal outcoupling structures have high cost as well as complex fabrication process and induce rough interfaces, which cause damage in organic layers and electrical shorts in devices. On the other hand, external outcoupling structures do not deteriorate the electrical functions of devices and can be readily introduced to the backside of substrate as a form of films. For external outcoupling, corrugated device structures [15,17], microlens array films [18–23], and micro-sphere scattering layers [16,24] have been extensively used. However, these external outcoupling structures typically need complex and expensive lithography process to form micro-shape patterns. White OLEDs with microlenses demonstrated by Sun et al. exhibited a 49% enhancement in external quantum efficiency (EQE) [19]. Koh et al. reported the down conversion white OLEDs with microlens arrays improving an EQE by 50% [25]. The microstructures on top electrodes for top-emitting OLEDs demonstrated by Liu et al. showed the 33% enhancement in the current efficiency of devices [26]. The breath figure methods have attracted much attention to prepare honeycomb microstructured patterns by a simple solution process. In the breath figure patterning, water droplets condensed on polymer surfaces produce ordered pore arrays on the polymer films after solvent and water evaporation [27,28]. Galeotti et al. reported an efficiency enhancement up to 34% for single-color OLEDs with microlens array films using breath figure techniques [20].

Together with enhancing outcoupling efficiency, a high quality of white light is of great importance for lighting applications. To realize white OLEDs, a most common approach is to stack red, green, and blue emissive layers in the device [1,29]. However, the stacked white OLEDs suffer from fabrication complexity, difficulty in solution process, and color-dependent lifetime [1,29,30]. Using down-conversion materials with blue-emitting OLEDs is an alternative approach [31–33], which is widely used in inorganic white light-emitting diodes based on yttrium aluminum garnet phosphors and GaN diodes. Part of blue light emitted from devices is converted to yellow, green, or red light by color-conversion materials attached in the backside of substrate, which is regarded as a cheap and simple approach to obtain white emission. However, the down-conversion techniques typically lead to the low device efficiency compared to stacked white OLEDs due to the light absorption loss from phosphors and limited performance of blue OLEDs, suggesting that light outcoupling techniques are of great necessity to realize the high-efficiency down-conversion white OLEDs. The development of the highly efficient blue emitters can offer enormous potential for high-performance white OLEDs combined with the down-conversion approaches.

In this work, we develop high-performance down-conversion microlens array (DC-MLA) films for down-conversion white OLEDs. The DC-MLA films are fabricated by a soft imprinting method with breath figure patterns. The breath figure patterns are obtained by simply directly dropping solvent on commercial polystyrene petri dishes in humid atmosphere, enabling low-cost, simple, and quick solution process, whereas conventional breath figure techniques typically need casting process of polymer films on the external substrates. White OLEDs employing the DC-MLA films exhibit the highly enhanced light outcoupling efficiency and the high quality of white light. The DC-MLA films improve the EQE and power efficiency of white OLEDs by a factor of 1.35 and 1.86, respectively. In addition, high-quality white light with a color-rendering index (CRI) of 84.3 is achieved for the white OLEDs with DC-MLA films. To the best of our knowledge, the outcoupling-enhanced white OLEDs show one of the highest enhancement factors reported for down-conversion white OLEDs with breath figure outcoupling patterns. We expect that the novel solution-processable DC-MLA films based on breath figure patterns can be a simple, cost-effective approach to realize high-performance down-conversion white OLEDs.

## 2. Experimental details

**Figure 1.**
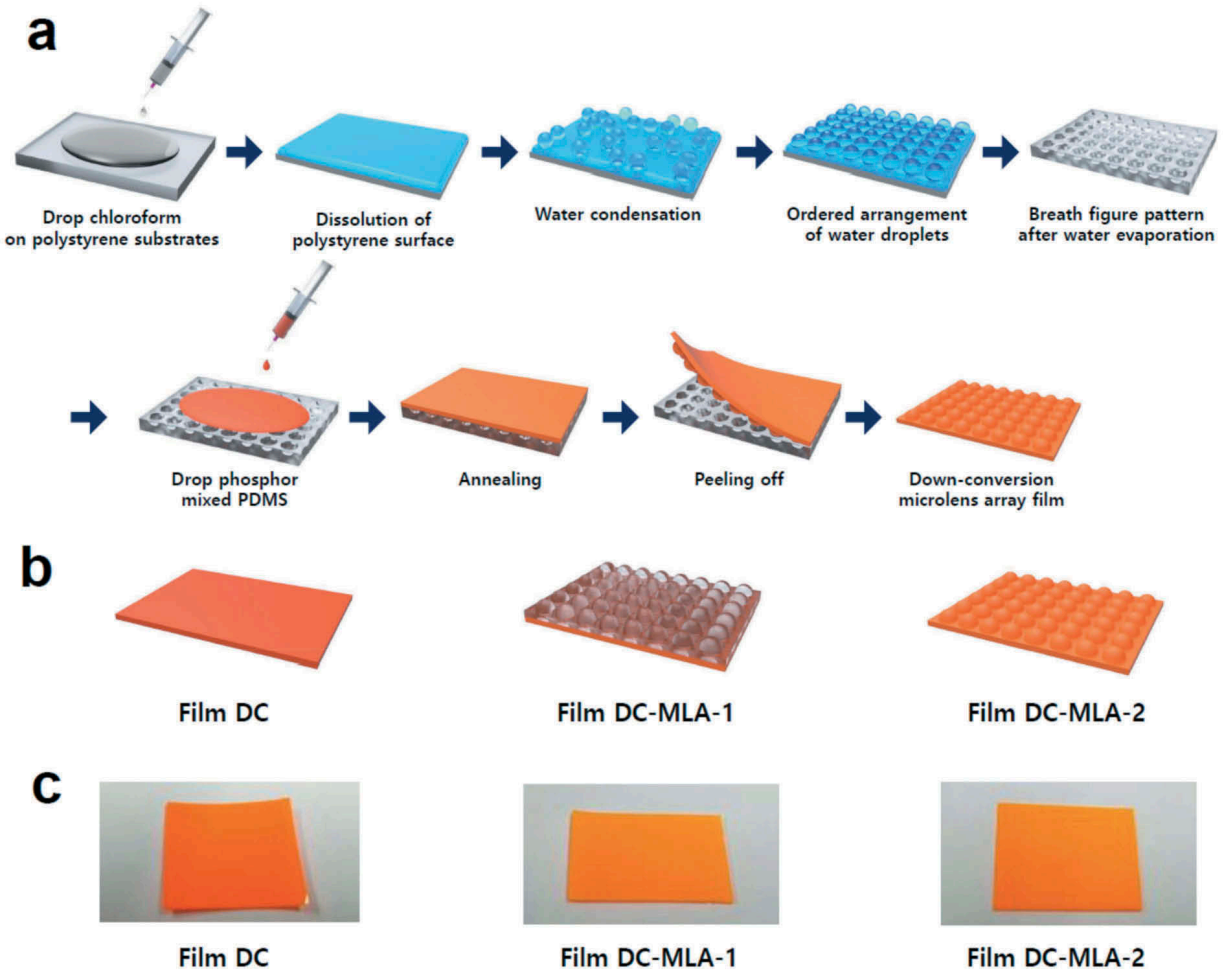
(a) Schematic illustration of the procedure for fabricating DC-MLA films using the polystyrene micro-patterned mold made by the breath figure method. (b) Structures and (c) photographs of Film DC, Film DC-MLA-1, and Film DC-MLA-2, respectively.

*Fabrication of down-conversion light outcoupling films*: The mold for the soft micro-pattern lithography is prepared by the breath figure method, which is a self-assembly-based method for manufacturing three-dimensional microscale patterns such as honeycomb-structured porous polymer films. The fabrication process of DC-MLA films is shown in Figure 1(a). As a first step, 1 ml of chloroform is dropped on commercial polystyrene petri dishes in humid atmosphere, where the films are subsequently kept for 60 min, resulting in the dissolution of the surface of polystyrene substrates. By evaporative cooling where the surface temperature is lower than dew-point temperature, water droplets condense on surfaces gradually, subsequently forming spontaneous hexagonal close-packed arrays of water droplets on the surface of polystyrene substrates. During the process, the water droplets sink into dissolved polystyrene solutions. The interfacial forces between the water droplets and the polymer solution have a strong influence on the breath figure patterns. After the completion of solvent evaporation from the polymer solution, ordered microporous patterns are formed on the polystyrene substrates [28,34]. The microporous polystyrene substrates prepared by the breath figure method serve as molds for forming microlens patterns on polydimethylsiloxane (PDMS).

The PDMS solutions (Sylgard 184, Dow Corning) were mixed with red (Sr_2_Si_5_N_8_:Eu^2+^) and green (Lu_3_Al_5_O_12_:Ce^3+^, LuAG) phosphors and subsequently sonicated for dispersing them. The phosphor-containing PDMS solutions were spin-coated on the glass substrates (Film DC) or on the microlens-patterned molds fabricated with the breath figure method (Film DC-MLA-2). Subsequently, PDMS films were cured at 70 °C for 1 h and the cured films were peeled off from the breath figure mold. For the preparation of Film DC-MLA-1, pure PDMS solutions without phosphors were spin-coated on the breath figure mold and were cured subsequently. The PDMS solutions mixed with phosphors were spin-coated on top of the cured PDMS films with microlens arrays and were peeled off from mold after curing. The fabricated down-conversion films were cut into rectangles and were applied on the glass substrates of blue OLEDs. The surface morphology and thickness of down-conversion films were examined by scanning electron microscopy (SEM, S-2400 Hitachi).

*Fabrication of devices*: OLEDs were fabricated by thermal evaporation onto prestructured indium tin oxide (ITO)-coated glass in a high vacuum chamber with a base pressure of approximately 10^−8^ mbar. The device architecture was the following (bottom to top): ITO/5 nm 1,4,5,8,9,11-hexaazatriphenylene hexacarbonitrile/50 nm 1-bis[4-[*N*,*N*-di(4-tolyl)amino]phenyl]-cyclohexane/5 nm 4,4′,4″-tris(*N*-carbazolyl)-triphenylamine:iridium(III)bis(4,6-difluorophenyl)-pyridinato-*N*,C2′)picolinate (FIrpic) (7 wt%)/5 nm 2,6-bis(3-(carbazol-9-yl)phenyl) pyridine (26DCzPPy):FIrpic (10 wt%)/55 nm 1,3-bis(3,5-dipyrid-3-yl-phenyl)benzene (BmPyPB)/30 nm BmPyPB:Li/1 nm LiF/100 nm Al. After processing, devices were immediately encapsulated in nitrogen atmosphere using a glass lid with an epoxy glue. The active area of devices was 2 × 2 mm^2^. The current–voltage–luminescence characteristics, angular luminance, and electroluminescence spectra were measured with a source-measure unit system (Keithley 238) and a goniometer-equipped spectroradiometer (Minolta CS-2000). The CRI of OLEDs was characterized by plugging spectra into a simulation software (Octave).

## 3. Results and discussion

For the fabrication of light outcoupling films with three-dimensional microlens structures, PDMS solutions are spin-coated on microporous polystyrene templates and are cured and peeled off subsequently. Finally, three kinds of down-conversion films are prepared using imprint techniques based on breath figure patterns as shown in Figure 1(b,c). The down-conversion film without outcoupling structures, e.g. flat PDMS with phosphors, is denoted as Film DC. The breath figure-patterned PDMS without phosphors combined with flat Film DC is denoted as Film DC-MLA-1. The phosphor-mixed PDMS directly imprinted by the breath figure mold is denoted as Film DC-MLA-2.

**Figure 2.**
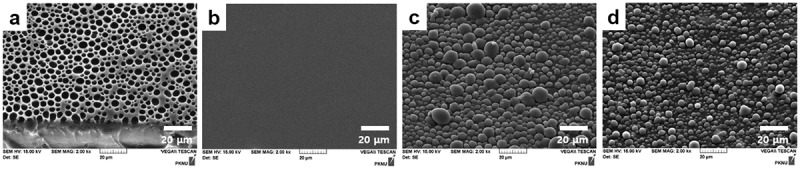
SEM images of (a) the microporous polystyrene mold made by the breath figure method, (b) the flat down-conversion film without phosphors (Film DC), (c) Film DC-MLA-1, and (d) Film DC-MLA-2. Both DC-MLA-1 and DC-MLA-2 are down-conversion microlens array films imprinted by the microporous polystyrene mold.

Figure 2 exhibits SEM images of porous polystyrene templates with breath figure patterns (Figure 2(a)) and various down-conversion films (Figure 2(b–d)). The breath figure-patterned polystyrene film shows compact pores with a size of several micrometers. Film DC shows flat surface, indicating that phosphors are successfully incorporated inside of PDMS films without surface bumps as shown in Figure 2(b). Both Film DC-MLA-1 and Film DC-MLA-2 exhibit well-developed outcoupling structures in the size of about 10–20 μm imprinted by microlens patterns of breath figure molds (Figure 2(c,d)). Individual sizes of microlens arrays for Film DC-MLA-2 are relatively smaller than that of Film DC-MLA-1. This might be caused by the slightly disturbed imprinting process at the interface between the micro-patterned mold and the PDMS film by the large particle size for the phosphors incorporated in Film DC-MLA-2 (5–25 μm for red and 10–50 μm for green phosphors). The disturbance of imprinting process by incorporated phosphors in PDMS is also observed in our previous study [33].

**Figure 3.**
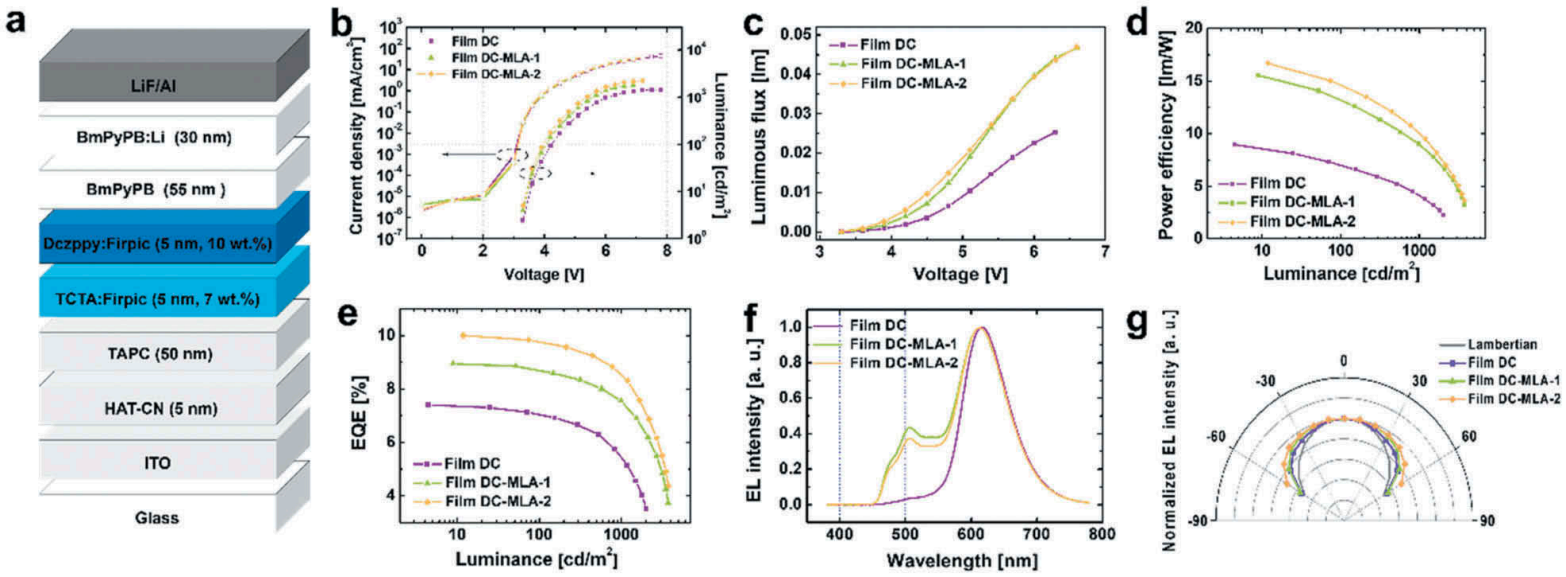
(a) Device structure for blue OLED. (b) Current–voltage–luminance curve, (c) luminous flux–voltage curve, (d) power efficiency, (e) EQE, (f) electroluminescence (EL) spectra, and (e) angular distribution of luminance for OLEDs with Film DC, Film DC-MLA-1, and Film DC-MLA-2, respectively.

To figure out the light outcoupling effects of down-conversion films on the efficiency of OLEDs, we employed Film DC, Film DC-MLA-1, and Film DC-MLA-2 into blue-emitting OLEDs. Note that any bump of films and air gap between PDMS films and glass substrates do not occur, thanks to the excellent elastomeric property of PDMS films. The structure of OLEDs is shown in Figure 3(a). For reliable investigation, we utilize the down-conversion films in blue OLED with a different architecture, which does not have an additional electron transport layer of BmPyPB:Li where a different cavity effect is expected (see Supplementary Information). It should be noted that the electrical properties of all devices are almost identical (see Figure 3(b)), ensuring reliable device fabrication process, since the external outcoupling films do not affect on the electrical properties of devices. The devices with both DC-MLA films show the higher luminance compared to OLED with Film DC at given voltages while electrical properties are almost equal (Figure 3(b,c)). This result clearly illustrates the effect of outcoupling enhancement for Film DC-MLA-1 and Film DC-MLA-2. Figure 3(d,e) show the power efficiencies and EQEs of OLEDs, respectively, and values are summarized in Table 1. By introducing Film DC-MLA-1 and Film DC-MLA-2, the EQEs of OLEDs are improved by a factor of 1.21 and 1.35, respectively, compared to that of OLED with Film DC. The EQE of Film DC-MLA-1 and Film DC-MLA-2-based OLEDs reaches 1.21% (15.56 lm/W) and 1.35% (16.70 lm/W), respectively. The power efficiencies of devices with Film DC-MLA-1 and Film DC-MLA-2 are improved by a factor of 1.73 and 1.86, respectively. It is observed that the outcoupling performance of OLEDs with Film DC-MLA-2 is higher than that of OLEDs with Film DC-MLA-1 despite the slightly smaller size of microlens arrays. The lower performance of OLEDs with Film DC-MLA-1 might be caused by a non-uniform film thickness or particle distribution attributed to the strong hydrophobicity of PDMS.

**Table 1.** Device performance of OLEDs based on down-conversion films.

	Film DC	Film DC-MLA-1	Film DC-MLA-2
Power efficiency (max) (lm/W)	8.97	15.56	16.70
EQE (max) (%)	7.41	8.95	10.01
Enhancement ratio (power efficiency)	–	1.73	1.86
Enhancement ratio (EQE)	–	1.21	1.35
CRI	57.2	84.3	83.7

CRI: Color-rendering index; EQE: external quantum efficiency; DC-MLA: down-conversion microlens array.

**Figure 4.**
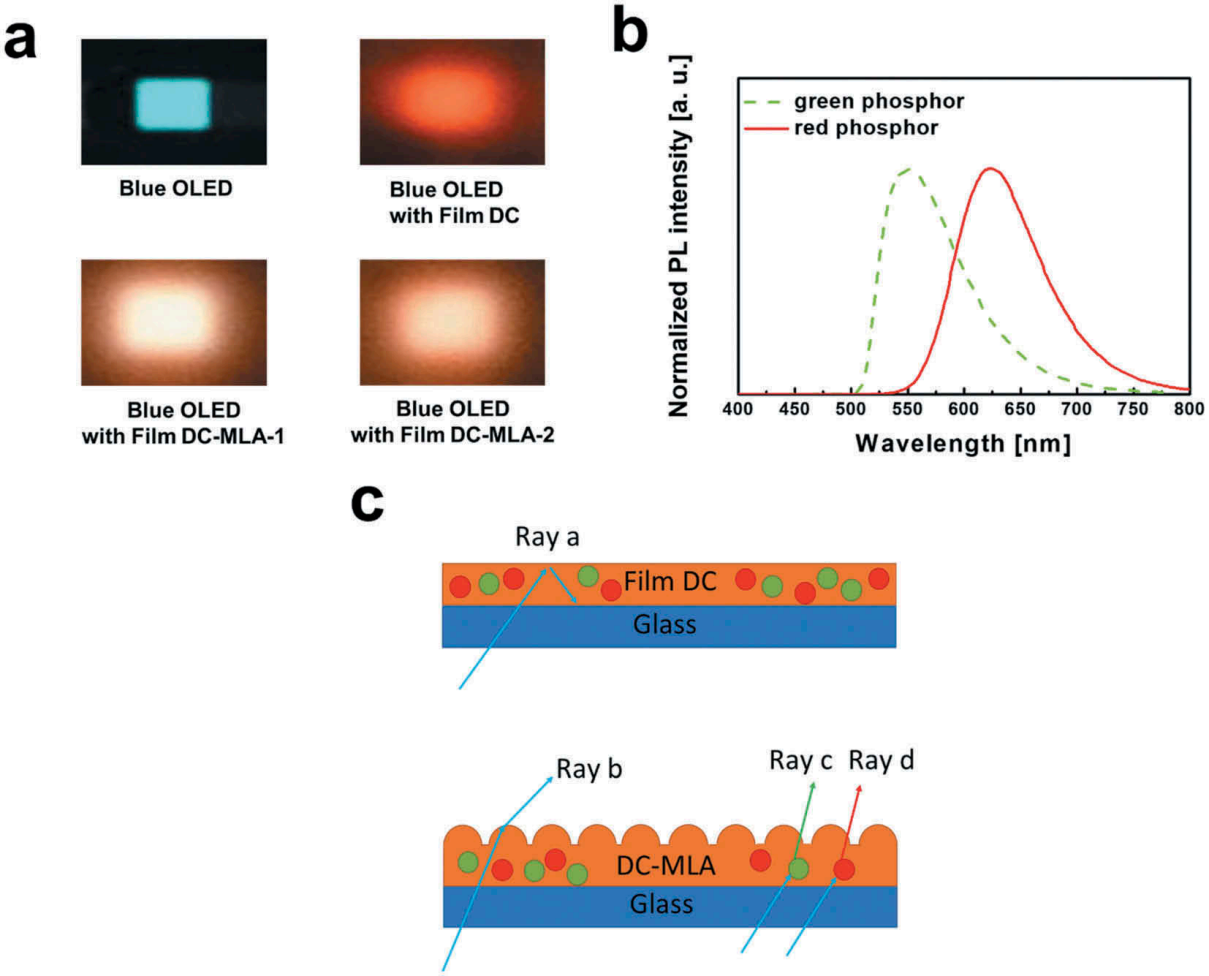
(a) Photographs of blue OLEDs and down-conversion white OLEDs with various down-conversion films in operation. (b) Normalized photoluminescence intensities of green and red phosphors. (c) Schematic sketch of rays in down-conversion films.

As can be seen in Figure 3(f), Film DC results in a very low light intensity in the blue region while Film DC-MLA-1 and Film DC-MLA-2 enhance the intensity of blue light. The imperfect down-conversion performance of Film DC can be also seen with the naked eye as shown in Figure 4(a). In contrast, the DC-MLA films successfully convert blue-emitted light to white light (Figures 3(f) and 4(a)). These results imply that rest of blue light is highly scattered inside of Film DC so that it cannot escape from Film DC which has a flat surface (Ray a in Figure 4(c)) while certain portion of blue light is absorbed in red and green phosphors for color conversion. The strong red emission indicates that absorbed blue light is mostly converted to red light. In contrast, unabsorbed blue light scattered from phosphors in Film DC-MLA-1 and Film DC-MLA-2 can be effectively extracted from the down-conversion films (Ray b in Figure 4(c)) due to the outcoupling structures formed by breath figure patterns which also extract the color-converted green and red light (Ray c and d in Figure 4(c)). Additionally, the enhancement of outcoupling in blue light leads to the significant improvement of the CRI of devices. The highest CRI of 84.3 is obtained for OLED with Film DC-MLA-1 while Film DC results in a CRI of only 57.2. The performance can be further enhanced by optimization processes such as the size control of microstructures or the thickness control of films supported by an optical simulation, which will be further studied.

The angular dependence of normalized luminance OLEDs with various kinds of down-conversion films is shown in Figure 3(g). All devices exhibit the higher luminance from an ideal Lambertian emission pattern and each shows slightly different angular dependence characteristics. For higher viewing angles above 30°, OLED with Film DC-MLA-2 has the higher normalized luminance than the devices with Film DC-MLA-1 and Film DC. The lowest normalized luminance is obtained for the device with Film DC, which does not have outcoupling structures.

## 4. Conclusions

We present down-conversion external outcoupling films with a high outcoupling efficiency and a high quality of white light for white OLEDs. The DC-MLA films are prepared by a simple imprinting process based on breath figure patterns. The EQE and the power efficiency of OLEDs are improved by a factor of 1.35 and 1.86, respectively, with Film DC-MLA-2. To the best of our knowledge, these are the highest enhancement factors reported for down-conversion white OLEDs with breath figure outcoupling patterns. In addition, DC-MLA films achieve the high-quality white light with a CRI of 84.3. The excellent outcoupling efficiencies and the high CRI of devices with DC-MLA films are caused by the outcoupling enhancement of scattered blue light inside of down-conversion films. We believe that the DC-MLA films prepared by simple imprinting techniques with breath figure patterns can be one of the most promising external outcoupling technologies for high-performance OLEDs.

## Supplementary Material

Supplemental Material
