# Supplementary material for: Enhanced outcoupling in down-conversion white organic light-emitting diodes using imprinted microlens array films with breath figure patterns
Source: Sci Technol Adv Mater. 2018 Nov 29;20(1):35–41. doi: 10.1080/14686996.2018.1551040 (PMC6346723; doi:10.1080/14686996.2018.1551040)
Supplement: Supplemental Material [file TSTA_A_1551040_SM2900.doc]

**Supplementary Information**

Enhanced Outcoupling in Down-Conversion White Organic Light-Emitting Diodes Using Imprinted Microlens Array Films with Breath Figure Patterns

Joo Won Han^1, †^, Chul Woong Joo^2, †^, Jonghee Lee^3,†^, Dong Jin Lee^1^, Jisoo Kang^4^, Seunggun Yu^5^, Woo Jin Sung^2^, Nam Sung Cho^2^, and Yong Hyun Kim^1,*^

^1^Department of Display Engineering, Pukyong National University, Busan 48513, Republic of Korea

^2^Flexible Information Device Research Center, Electronics and Telecommunications Research Institute (ETRI), Daejeon 34129, Republic of Korea

^3^Department of Creative Convergence Engineering, Hanbat National University, Daejon 34158, Republic of Korea

^4^Robert Frederick Smith School of Chemical and Biomolecular Engineering, Cornell University, New York 14853, United States

^5^Department of Materials Science and Engineering, Yonsei University, Seoul 03722, Republic of Korea

^†^These authors contributed equally to this work

*Corresponding authors

E-mail: yhkim113@pknu.ac.kr

Tel. : +82-51-629-6418; Fax : +82-51-629-6408

**Figure S1.** (a) Device structures for blue OLED, which does not have an additional electron transport layer of BmPyPB:Li. (b) Current-voltage-luminance curves, (c) power efficiency, (d) electroluminescence spectra, and (e) angular distribution of luminance of OLEDs with Film DC, Film DC-MLA-1, and Film DC-MLA-2, respectively.

**Figure S2.** External quantum efficiency (EQE) of OLEDs without a BmPyPB:Li layer based on down-conversion films. The imperfect structure of Film DC-MLA-1 may cause the deteriorated outcoupling performance for the device without BmPyPB:Li, compared to the device with BmPyPB:Li. The different cavity effect from both devices also gives a different enhancement ratio.


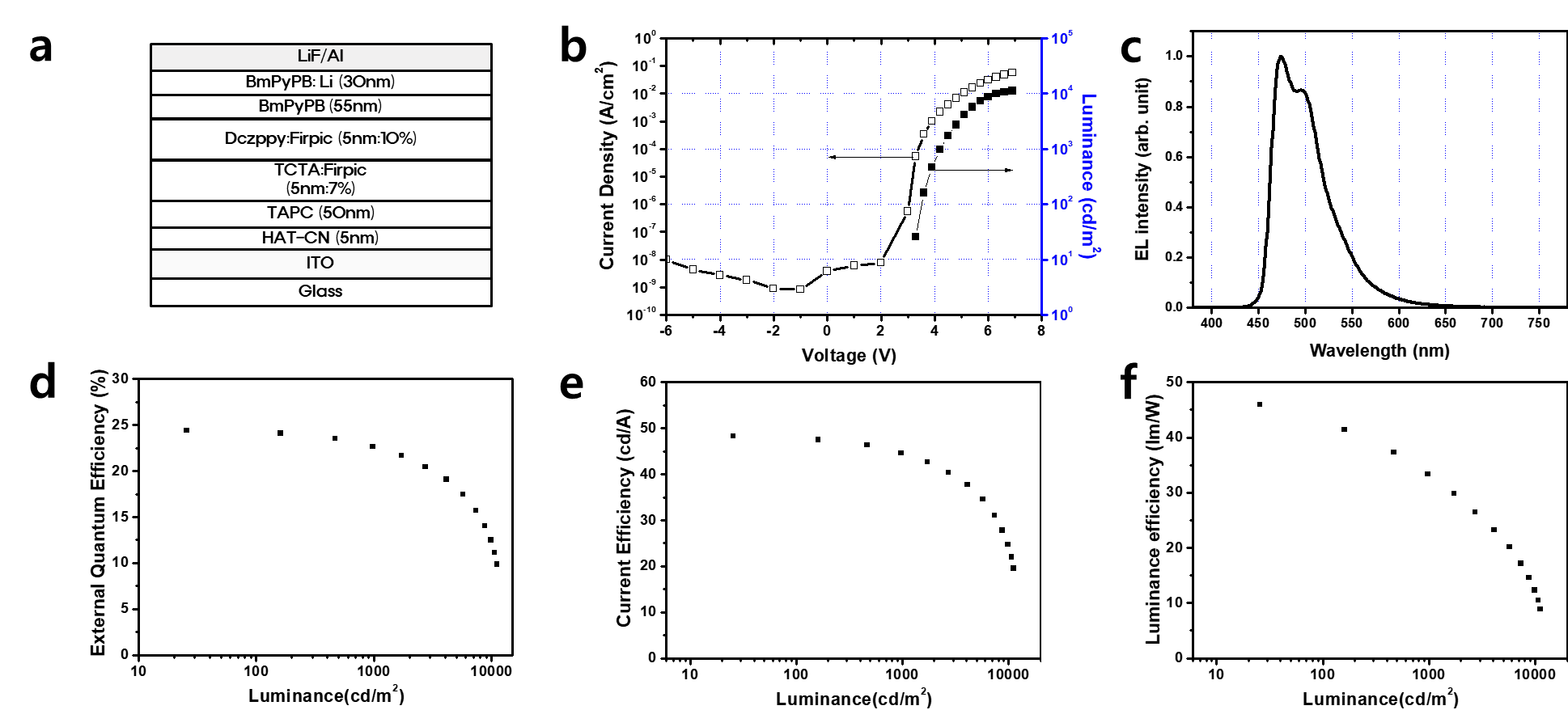


**Figure S3.** (a) Device structures for blue OLED. (b) Current-voltage-luminance curves, (c) electroluminescence spectrum, (d) EQE, (e) current efficienct, and (f) power efficiency of OLEDs without the DC-MLA film, respectively.

|  | Film DC | Film DC-MLA-1 | Film DC-MLA-2 |
| --- | --- | --- | --- |
| Power efficiency (Max.) [lm/W] | 7.49 | 11.4 | 13.58 |
| Enhancement ratio [%] | - | 1.52 | 1.81 |
| CRI | 44.9 | 75.5 | 77.4 |

**Table S1**. Device performance of OLEDs without a BmPyPB:Li layer based on down-conversion films.
